# Challenges of the Regional Anesthetic Techniques in Intensive Care Units – A Narrative Review

**DOI:** 10.2478/jccm-2024-0023

**Published:** 2024-07-31

**Authors:** Alexandra Elena Lazar, Mihaela Butiulca, Lenard Farczadi

**Affiliations:** George Emil Palade University of Medicine, Pharmacy, Science, and Technology of Targu Mures, Romania; Center for Advanced Medical and Pharmaceutical Research, George Emil Palade University of Medicine, Pharmacy, Science, and Technology of Targu Mures, Romania

**Keywords:** regional techniques, critically ill. intensive care, anticoagulation, pain

## Abstract

Effective pain management is vital for critically ill patients, particularly post-surgery or trauma, as it can mitigate the stress response and positively influence morbidity and mortality rates. The suboptimal treatment of pain in Intensive Care Unit (ICU) patients is often due to a lack of education, apprehensions about side effects, and improper use of medications. Hence, the engagement of pain management and anesthesiology experts is often necessary.

While opioids have been traditionally used in pain management, their side effects make them less appealing. Local anesthetics, typically used for anesthesia and analgesia in surgical procedures, have carved out a unique and crucial role in managing pain and other conditions in critically ill patients. This work aims to offer a comprehensive overview of the role, advantages, challenges, and evolving practices related to the use of local anesthetics in ICUs. The ability to administer local anesthetics continuously makes them a suitable choice for controlling pain in the upper and lower extremities, with fewer side effects.

Epidural analgesia is likely the most used regional analgesic technique in the ICU setting. It is primarily indicated for major abdominal and thoracic surgeries, trauma, and oncology patients. However, it has contraindications and complications, so its use must be carefully weighed. Numerous challenges exist regarding critically ill patients, including renal and hepatic failure, sepsis, uremia, and the use of anticoagulation therapy, which affect the use of regional anesthesia for pain management. Appropriate timing and indication are crucial to maximizing the benefits of these methods.

The advent of new technologies, such as ultrasonography, has improved the safety and effectiveness of neuraxial and peripheral nerve blocks, making them feasible options even for heavily sedated patients in ICUs.

## Introduction

The use of local anesthetics in Intensive Care Units (ICUs) represents a critical aspect of modern critical care medicine. Intensive care experts are becoming more essential in preventing and treating both physical and psychological stress in critically ill patients. This is crucial to avoid harmful outcomes like systemic inflammatory response syndrome, cardiac issues, and posttraumatic stress disorder [1]. Nonetheless, the role of pain relief in adequate stress management is not thoroughly discussed, and existing guidelines, largely derived from personal clinical experiences, are limited.

Considering the adverse effects of opioids, which include respiratory depression, altered mental states, and decreased bowel movement and withdrawal symptoms, regional analgesia through neuraxial and peripheral nerve blocks presents considerable benefits [2, 3]. A major challenge in providing effective pain relief for critically ill patients is the absence of a universally accepted pain assessment tool, or “pain meter”. Many patients in critical care units are unable to communicate or use standard visual or numeric scales for pain measurement. Alternative tools, adapted from pediatric or geriatric practices, focus on facial expressions and other physical reactions to pain. These may be valuable, but their effectiveness in the ICU remains under-researched. Indirect indicators of pain, such as changes in heart rate and blood pressure during routine care activities, dressing changes, or wound care, can be useful [4]. Additionally, sedation scales like the Ramsay Sedation Scale or the Riker Sedation-Agitation Scale may offer some insights, though not originally intended for pain assessment [5].

Local anesthetics, traditionally used for anesthesia and analgesia in surgical settings, have found a unique and vital place in the management of pain and other conditions in critically ill patients. This work provides an overview of the role, benefits, challenges, and evolving practices surrounding the use of local anesthetics in ICUs.

## Pain in ICU

Pain in ICU is still a challenge for the intensivists. It is a known fact that everybody acknowledges its existence and its side effects, but we have yet to create a modality to quantify it and personalize its treatment.

The International Association for the Study of Pain defines pain as an “unpleasant sensory and emotional experience associated with actual or potential tissue damage or described in terms of such damage” [6].

Critically ill patients pose supplementary challenges in pain diagnosis because most of the patients cannot verbalize their pain Intensive care unit, and to date, the best pain evaluator is the patient. This discomfort can stem from various causes such as surgery, trauma, burns, or cancer. All critically ill adults in ICUs, including those in medical, surgical, and trauma units, often suffer from pain, whether at rest or during routine ICU procedures. They have the right to proper pain management [7]. Pain triggers an acute stress response, which can lead to alterations in the neurovegetative system and neuroendocrine release, and can often result in psychological effects like agitation [8, 9].

Pain management is often inadequate due to concerns about reducing spontaneous breathing, causing opioid addiction, and causing cardiovascular instability. Additionally, there’s a lack of clear understanding among many healthcare providers about how to assess pain accurately, treat it effectively, and recognize the long-term advantages of proper pain management. Effective pain management involves not just lessening the severity of pain but also enhancing the patient’s functional capacity. Reducing opioid-related side effects like nausea, vomiting, urinary retention, and drowsiness can aid in the patient’s recovery and potentially reduce the duration of their stay in the ICU and hospital [10].

## The Role of Local Anesthetics in ICUs

Local anesthetics work by blocking sodium channels, which in turn prevents the propagation of nerve impulses, thereby offering a localized loss of sensation, including pain [11]. In ICUs, the use of these agents is not only limited to procedural analgesia but extends to the management of chronic pain, post-operative care, and adjuncts in the treatment of certain medical conditions. For instance, lidocaine, a common local anesthetic, has been used intravenously for treating conditions like ventricular arrhythmias [12].

In the ICU setting, their use is multifaceted, ranging from minor procedures such as insertion of catheters to more complex applications like post-surgical pain management or treatment of chronic conditions such as neuropathic pain.

One of the primary benefits of local anesthetics in the ICU is their ability to target pain at its source without causing systemic side effects. Unlike systemic analgesics, which can affect the entire body and often carry risks of sedation, respiratory depression, or even addiction if we think about major analgesics like opioids, local anesthetics provide a safer alternative, especially for patients with complex medical conditions [13].

Another critical aspect of local anesthetics in the ICU is their role in facilitating patient recovery and rehabilitation. Effective pain management enables patients to participate more actively in physiotherapy and other rehabilitation activities, which is crucial for their recovery process. Additionally, by alleviating pain, local anesthetics can help reduce the stress response associated with acute pain, potentially shortening the ICU stay and improving long-term outcomes [14].

However, the use of local anesthetics is not without challenges. Precise dosing is crucial, as too high a dose can lead to toxicity, while too low a dose may be ineffective. Moreover, the administration of local anesthetics requires skilled personnel and careful monitoring, especially in patients with compromised cardiovascular or neurological functions.

## Application and Techniques

### Upper extremity blocks

Currently, there is a lack of randomized, controlled studies or extensive prospective research on the application of peripheral nerve blocks for upper extremity injuries in critically ill patients. However, serious injuries to the shoulder or arm are often seen in patients with multiple traumas, and these injuries are frequently accompanied by blunt chest trauma that necessitates mechanical ventilation. These types of injuries can lead to significant pain, particularly during patient repositioning. In cases where the orthopedic trauma is part of a more complex injury that includes brain damage, affecting the patient’s mental status, traditional opioid-based pain relief methods might obscure neurological assessments. In such scenarios, adequate pain management for the shoulder or upper limb can be effectively accomplished through continuous techniques like interscalene, cervical paravertebral, or infraclavicular approaches targeting the brachial plexus.

Concerns are particularly heightened when considering the administration of regional blocks in ICU patients who have compromised mental status, either due to neurological injuries or the effects of therapeutic sedation. Benumof’s report of a small series highlighted severe complications, including spinal cord injuries, linked to the interscalene approach, potentially connected with the use of sedation or general anesthesia. In these cases, spinal cord injuries were associated with patients under heavy sedation or anesthesia, rather than direct injury to the peripheral nerves [15].

Crucially, the execution of such blocks should be reserved for healthcare professionals who possess the necessary experience. One must consider the inevitable impact on the phrenic nerve and the consequent loss of function in half of the diaphragm when planning such procedures. While the blockage of the phrenic nerve typically has minimal impact on patients who are mechanically ventilated, it can pose challenges in weaning high-risk patients off mechanical ventilation. Additionally, the risk of infection may be heightened due to the proximity of the interscalene catheter’s insertion site to a tracheostomy tube, necessitating vigilant and standardized monitoring of the insertion site. Furthermore, difficulties in positioning can restrict the use of the cervical paravertebral approach, despite its effectiveness in providing pain relief for the shoulder, arm, and hand [16].

The continuous infraclavicular and axillary methods are effective in providing pain relief for most of the arm, elbow, and hand. In patients requiring surgical anesthesia for procedures like painful dressing changes or debridement in cases of burns or extensive soft tissue wounds, administering a bolus of local anesthetic via the catheter should be considered. The lateral infraclavicular approach is advantageous as it reduces the risk of pneumothorax and allows for better stabilization of the catheter. This contrasts with more proximal brachial plexus block techniques, where the catheter is placed in a more superficial position in softer, more mobile tissue [16, 17].

Nerve blocks for the lower extremities Femoral nerve catheters prove to be effective for controlling severe pain stemming from fractures of the femoral neck, from the time of injury until just after the fracture has been surgically stabilized. The expert application of ultrasound can minimize the pain typically associated with nerve stimulation in such cases. This pain can also be managed with low doses of intravenous remifentanil (0.3–0.5 mcg/kg) or ketamine (0.2–0.4 mg/kg). An alternative technique is the fascia iliaca compartment block [17].

For comprehensive pain relief of the entire leg, and even to serve as surgical anesthesia for procedures such as external fixation, a continuous femoral catheter can be used in conjunction with a sciatic nerve block. The choice between an anterior or posterior approach to the sciatic nerve, with one or two injections, is largely dependent on the practitioner’s expertise and the patient’s capacity to be positioned properly for the block [16].

### Paravertebral and erector spinae blocks in critically ill

Erector spinae plane block (ESPB) and thoracic paravertebral block (TPVB) are both utilized for postoperative analgesia but have distinct applications and challenges, particularly in critically ill patients.

For posterolateral rib fractures, a study highlighted the efficacy of both ESPB and TPVB in managing pain, with ESPB demonstrating a significant reduction in 48-hour fentanyl consumption compared to EPVB. The visual analog scale (VAS) scores were better in the thoracic epidural analgesia (TEA) group at various time points, indicating superior pain control. However, ESPB is noted for its simplicity and lower risk profile, including reduced chances of pneumothorax and easier administration, especially in patients on anticoagulant therapy which is a contraindication for TPVB and TEA [17].

A randomized clinical trial comparing ESPB and PVB for postoperative analgesia after breast surgery found that patients receiving PVBs experienced superior analgesia and reduced opioid requirements compared to those receiving ESPBs. This suggests that while ESPB has a favorable safety profile and ease of administration, PVB may provide more effective pain control in specific surgical contexts [18].

Another study on postoperative analgesia after video-assisted thoracic surgery found no significant difference in pain severity measured by VAS scores between ESPB and TPVB groups, indicating comparable effectiveness. This supports the potential of ESPB as a viable alternative to TPVB, offering benefits such as ease of administration and a favorable safety profile, especially in critically ill or anticoagulated patients [19].

In the context of critically ill patients, these findings suggest that ESPB offers a valuable alternative to TPVB, especially considering its ease of administration, lower risk of complications, and applicability in patients where TPVB and TEA are contraindicated. However, the choice between ESPB and TPVB may depend on the specific clinical scenario, patient condition, and the need for unilateral versus bilateral pain control. Further research and larger, prospective studies may provide more definitive guidance on the optimal use of these blocks in various surgical and trauma settings.

## Challenges and Consideration in performing regional block in the critically ill

Despite their benefits, the use of local anesthetics in ICUs comes with challenges. Dosage and administration need careful consideration, as critically ill patients often have altered pharmacokinetics and pharmacodynamics. Overdosage or inappropriate administration can lead to systemic toxicity, manifesting as CNS and cardiovascular disturbances. Furthermore, patients in ICUs often have multiple comorbidities and are on various medications, raising concerns about drug interactions and contraindications.

Table 1 summarizes some of the indications, contraindications and practical problems of peripheral blocks in critically ill patients.

**Table 1.** Main limb blocks with their indications, contraindications, and practical concerns from S *Stubner Schulz: NYSORA, 2023* [23].

**Block**	**Indications**	**Contraindications**	**Practical Problems**
Interscalene	Shoulder/arm pain	Untreated contralateral pneumothorax	Horner syndrome Dependence on diaphragmatic breathing Contralateral vocal cord palsy Proximity to tracheostomy and jugular vein catheter insertion sites Local infection at puncture site
Cervical paravertebral	Shoulder/elbow/wrist pain	Severe coagulopathy	Horner syndrome may obscure neurologic assessment Dependence on diaphragmatic breathing Contralateral vocal cord palsy Block of ipsilateral phrenic nerve Local infection at the puncture site Patient positioning
Infraclavicular	Arm/hand pain	Severe coagulopathy Untreated contralateral pneumothorax	Pneumothorax risk Untreated contralateral pneumothorax Steep angle for catheter placement Interference with subclavian lines Local infection at puncture site
Axillary	Arm/hand pain	Local infection at puncture site	Arm positioning Catheter maintenance
Paravertebral Thoracic Lumbar	Unilateral chest or abdominal pain restricted to few dermatomes	Severe coagulopathy Untreated contralateral pneumothorax	Patient positioning Stimulation success sometimes hard to visualize Local infection at puncture site
Femoral or sciatic	Unilateral leg pain	Severe coagulopathy Local infection at puncture site	Patient positioning Interference of femoral nerve catheters with femoral lines

## Neuraxial blocks for the critically ill

### Epidural and Spinal Blocks

Epidural analgesia is probably the most used regional analgesic technique in the ICU setting. Some indications in which epidural analgesia may not improve mortality rates but may facilitate management and improve patient comfort in the ICU include chest trauma, thoracic and abdominal surgery, major vascular surgery, major orthopedic surgery, acute pancreatitis, paralytic ileus, cardiac surgery, intractable angina pain [20]. Although high-risk patients seem to profit most from epidural analgesia, the current literature does not address the specific circumstances of critically ill patients with multiple comorbidities and organ failure. For that reason, an individual approach is necessary when considering the application of epidural analgesia in this population.

In a survey covering 216 general ICUs in England, it was found by Low that 89% used epidural analgesia. However, only a third of these units had formal policies for its implementation. While 68% avoided placing epidural catheters in patients with confirmed blood infections, only half considered the absence of infection or systemic inflammatory response syndrome (SIRS) as a deterrent. The survey also indicated that issues such as lack of patient consent or the requirement for anticoagulants following catheter placement were not widely regarded as reasons to avoid epidural catheter insertion. In elective procedures, managing consent, potential bleeding disorders, and infection risks are relatively manageable, but these issues pose significant challenges in patients who are newly admitted with conditions like multiple traumas or severe intra-abdominal issues, including acute pancreatitis [21]. Furthermore, the practice of inserting epidural catheters in sedated patients is contentious and confirming correct catheter placement is difficult in critically ill patients when sensory level testing is unreliable.

The possibility of accidentally placing an epidural catheter in the intrathecal space always necessitates vigilant caution. The advent of novel laboratory methods, such as the beta 2 transferrin test, has become increasingly useful for identifying cases where an epidural catheter has been unintentionally inserted into the subarachnoid space [22].

Arranging the patient’s position for epidural procedures can be complicated, influenced by factors such as the type of injury, the arrangement and quantity of drains and catheters, and any external fixation devices in use. Table 2 offers an overview of the indications for epidural catheter use, the reasons to avoid it, and the practical issues encountered during its placement.

**Table 2.** Indications, contraindications and practical concerns for epidurals in critically ill patients – from *S Stubner Schulz: NYSORA, 2023* [23].

**Indications**	**Contraindications**	**Practical concerns**
Chest trauma	Coagulopathy or current use of anticoagulants during catheter placement and removal	Positioning of patient
Thoracic surgery		Monitoring of neurologic function (consider MEP/SSEP)
Abdominal surgery		
Paralytic ileus		
Pancreatitis	Sepsis/bacteremia	
Intractable angina	Local infection at the puncture site	
Orthopedic surgery or trauma of lower extremities	Severe hypovolemia, Acute hemodynamic instability	
Peripheral vascular disease of lower extremities	Obstructive ileus	

The role of experienced nursing staff is pivotal in ensuring the patient is positioned correctly and in the safe handling of tubes and catheters throughout the procedure. Adopting comprehensive barrier measures, like those employed for central line insertions, is recommended when inserting epidural catheters in critically ill patients. To minimize the likelihood of the catheter becoming dislodged and to lower the risk of infection at the catheter site, tunnelling the catheter should be considered. For confirming the catheter’s proper placement, using electrical stimulation during the insertion process or a subsequent X-ray with a minimal amount of safe contrast material might prove helpful [20].

Utilizing bolus doses of long-acting local anesthetics such as bupivacaine, ropivacaine, or levobupivacaine, or pausing the continuous infusion as required, facilitates neurological evaluations when needed. Monitoring techniques like motor-evoked potentials (MEPs) in the lower extremities and somatosensory-evoked potentials (SSEPs) of the tibial nerve can provide valuable insights for neurological assessments, particularly in cases where the patient’s compromised mental state hinders traditional examinations. Although this approach is standard in operating rooms for assessing spinal cord health and managing spinal cord injuries, its effectiveness in ICU settings with epidural analgesia has not been thoroughly evaluated [20]. All catheter techniques have the advantage of being included in a PCA (Patient Controlled Analgesia) where the patient can administer himself, at specific times and with specific dosages, analgesics.

The primary side effects associated with epidural blocks are typically bradycardia and hypotension, consequences of blocking the sympathetic nervous system. These hemodynamic changes are more pronounced when using intermittent bolus administration, in patients with low blood volume, and in those whose venous return is reduced due to high positive end-expiratory pressure (PEEP) in their ventilation.

## Special circumstances in the critically ill

The critically ill patient is a complex patient with multiple co-morbidities and treatments some of which may interfere with regional procedures – peripheral or central. Some of the situations have been summarized in Table 3 with the amendment that in their active stages, all these conditions may lead to considerable coagulopathy. When contemplating the use of regional anesthesia for postoperative pain relief or similar purposes, it is crucial to follow the guidelines for that respective situation.

**Table 3.** Special circumstances in critically ill and their consideration - from *W.Haroop 2013. Anest*.[35]

**Condition**	**Description**
**Trauma**	Triggered by factors such as tissue trauma, shock, dilution of blood components, low body temperature, increased acidity in the blood, and inflammation, it is advisable to evaluate the possibility of coagulopathy before proceeding with any regional anesthesia technique.
**Sepsis**	Severe sepsis often results in a state that promotes blood clotting. The use of preventive measures against deep vein thrombosis is endorsed in these cases. Septic shock may give rise to a type of coagulopathy characterized by the consumption of clotting factors. Due to the associated risks of epidural abscess and meningitis, systemic sepsis is generally considered a relative contraindication for certain procedures.
**Uremia**	Coagulopathy resulting from a low platelet count necessitates evaluating both the quantity and functionality of platelets. The administration of DDAVP can enhance platelet function. In patients with chronic kidney disease undergoing dialysis, it’s important to consider any remaining anticoagulant effects post-dialysis.
**Liver Failure**	The liver produces all clotting factors except for factor VIII. In cases of liver failure, it’s crucial to evaluate any disturbances in blood clotting. Issues like a reduced platelet count and impaired platelet function may occur. It’s essential to both assess and address any coagulation disorders in this context.
**Massive Transfusion**	Changes in blood clotting resulting from the dilution and depletion of clotting factors call for an evaluation of coagulopathy. This assessment is best conducted once bleeding has been managed and the patient has stabilized. Additionally, assessing platelet functionality is necessary after administering platelet transfusions.
**Disseminated Intravascular Coagulopathy**	The abnormal triggering of the blood clotting process results in a condition known as consumptive coagulopathy. In cases of Disseminated Intravascular Coagulopathy (DIC), performing neuraxial blockade is considered unsafe. Therefore, when considering peripheral blocks, they should be administered at sites where compression is feasible.

Drawing from early 20th-century observations of lumbar punctures and meningitis, present-day sepsis and bacteremia are viewed as contraindications for using intrathecal opioids and, similarly, for inserting an epidural catheter. Nonetheless, many patients in intensive care units (ICUs), particularly those recovering from trauma or significant surgeries, often exhibit symptoms of the Systemic Inflammatory Response Syndrome (SIRS). The presence of fever and a heightened white blood cell count, without positive blood culture results, does not conclusively indicate bacteremia [24].

The assessment of serum indicators like C-reactive protein (CRP), procalcitonin, and interleukin-6 together has proven effective in accurately detecting bacterial sepsis, aiding in decisions regarding the insertion of epidural catheters. For managing a patient’s coagulation status, it is important to follow the current guidelines set by the American Society of Regional Anesthesia and Pain Medicine (ASRA). Ensuring proper timing intervals for anticoagulant medication is crucial for the safe placement and removal of epidural catheters. Although there is no strong evidence linking coagulopathy or the use of therapeutic anticoagulants to an increased risk of epidural hematoma with an indwelling catheter, the potential for this severe complication should be carefully weighed against the advantages of epidural analgesia [25].

Apprehension regarding nerve damage is a common reason for avoiding peripheral nerve blocks in patients under heavy sedation. Nonetheless, it may be justifiable to proceed with regional analgesia blocks after a thorough evaluation of the risks and benefits, especially if the clinical circumstances deem it necessary [26].

The recent development of ultrasound-guided (USG) techniques has revolutionized the process, making it easier and more accurate to pinpoint neural structures. This leads to the safer use of lower amounts of local anesthetics and facilitates the placement of nerve catheters in ICU patients, even those under heavy sedation [26]. These techniques have shown high effectiveness, with reports of reduced time to achieve full analgesia. Furthermore, the precise needle placement enabled by ultrasound guidance means less medication is needed, a crucial advantage for critically ill patients who might require several blocks, such as those with multiple surgical sites or who have sustained multiple injuries [27].

## Coagulation abnormalities

The critically ill patients are usually on anticoagulation/antithrombotic medication for various reasons, hence this situation can impact all regional techniques performed on these patients, whether the technique is continuous or single shot.

Table 4 presents the guidelines for regional techniques and the most encountered anticoagulation drugs in ICUs.

**Table 4:** The most used anticoagulant drugs in ICU and their relation to regional techniques- *from W.Haroop 2013. Anest*. [35] - UFH, unfractionated heparin; sc, subcutaneous; APTTR, activated partial thromboplastin time ratio; iv, intravenous; LMWH, low molecular weight heparin, NSAIDs, non-steroidal anti-inflammatory drugs; INR, international normalized ratio; CrCl, creatinine clearance.

**Drug**	**Time to Peak Effect**	**Elimination Half-Life**	**Acceptable Time after Drug for Block Performance**	**Administration of Drug while Spinal or Epidural Catheter in Place**	**Acceptable Time after Block Performance or Catheter Removal for Next Drug Dose**
UFH sc prophylaxis	<30 min	1–2 h	4 h or normal APTTR	Caution by manufacturer	1 h
UFH iv treatment	<5 min	1–2 h	4 h or normal APTTR	Caution by manufacturer	24h
LMWH sc prophylaxis	3–4h	3–7 h	12 h	Caution by manufacturer	34h
LMWH sc treatment	3–4h	3–7 h	24 h	Not recommended	4 h
Danaparoid prophylaxis	4–5 h	24 h	Avoid (consider anti-Xa levels)	Not recommended	6 h
Danaparoid treatment	4–5 h	24 h	Avoid (consider anti-Xa levels)	Not recommended	6 h
Bivalirudin	5 min	25 min	10 h or normal APTTR	Not recommended	6 h
Argatroban	<30 min	30–35 min	4 h or normal APTTR	Not recommended	6 h
Fondaparinux prophylaxis	1–2h	17–20 h	36–42 h (consider anti-Xa levels)	Not recommended	6–12 h
Fondaparinux treatment	1–2h	17–20 h	Avoid (consider anti-Xa levels)	Not recommended	12 h
NSAIDs	1–12 h	1–12 h	No additional precautions	No additional precautions	No additional precautions
Aspirin	12–24 h	Not relevant; irreversible effect	No additional precautions	No additional precautions	No additional precautions
Clopidogrel	12–24 h	7 days	Not recommended	6 h	6 h
Prasugrel	15–30 min	7 days	Not recommended	6 h	6 h
Ticagrelor	2 h	8–12 h	5 days	Not recommended	6 h
Tirofiban	<5 min	4–8h	68 h	Not recommended	6 h

## Other regional techniques for critically ill patients

Often not remembered, yet simple and safe to perform, are single-injection nerve blocks like intercostal blocks for chest tube insertion, scalp blocks for halo device application, and sufficient local infiltration for typical ICU processes such as placing arterial and central venous catheters, conducting lumbar punctures, and performing ventriculostomies. For optimal effectiveness of EMLA cream as a topical anesthetic, it should be applied 30 to 45 minutes before the procedure. Instead of epidural catheters, intrathecal morphine injections, either as a one-time injection or through a spinal catheter, may be a suitable option, especially for brief post-surgical applications [28].

### Celiac plexus block

Celiac plexus blocks can be highly effective for managing pain associated with pancreatitis and cancer-related upper abdominal issues. However, their practicality for patients who are acutely and critically ill is limited due to the technical complexities involved, such as the requirement for computed tomography (CT) guidance, fluoroscopy, or trans gastric ultrasound, and the necessity for multiple injections [29].

### Intrapleural catheters

The use of intrapleural catheters for managing pain following chest trauma is somewhat restricted due to the simultaneous requirement for chest tube drainage. Additionally, the risk of pneumothorax reduces their effectiveness in pain management post-traditional cholecystectomy, especially when compared to epidural or paravertebral techniques in patients on ventilation. However, thoracic paravertebral catheters can be a useful substitute for epidural catheters in managing pain that is unilateral and confined to a limited number of dermatomes, such as in cases of rib fractures or zoster neuralgia [30].

### Transversus abdominal block (TAP)

This regional analgesia approach has been effective in providing post-surgical pain relief for various abdominal wall procedures. To decrease the risk of potential complications such as intraperitoneal injection leading to bowel injury or hematoma, liver laceration, temporary femoral nerve paralysis, accidental intravascular injection, infection, and catheter breakage, the use of a fine-gauge, blunt-tipped, short bevel needle along with ultrasound guidance (USG) is recommended. Additionally, USG techniques enable targeted blocking of either the upper or lower parts of the abdominal wall [31,32,33,34].

## Pain measurement devices

There are several innovative devices on the market designed to measure pain, each using different methods to quantify pain levels objectively. These devices range from wearable sensors to handheld units and even include methods that assess pain through brain activity.

1. eEgg: The eEgg device measures pain intensity and compares it with standard methods like the numeric rating scale (NRS). It translates pain sensations into measurable handgrip strength values and has shown good correlations in validity and reliability with the hand dynamometer. [36].
2. fNIRS System: Developed by MIT researchers, this device detects pain levels by analyzing brain activity, specifically changes in oxygenated and deoxygenated hemoglobin in the prefrontal cortex. It employs machine learning to improve accuracy and adaptability to individual pain responses [37].
3. AlgometRx: This handheld device measures a patient’s pupillary response to pain and uses proprietary algorithms to provide a diagnostic measurement of pain intensity and type. It aims to offer a more objective way to assess pain, particularly useful for pediatric and non-verbal patients [38–39].
4. PainQx: A platform that utilizes an EEG headset to map out a patient’s pain reaction by studying electroactivity data. It aims to provide an objective measure of pain levels to potentially improve treatment and management [40].
5. Electrical Stimulation and Pressure Device: Developed for a pilot study, this device uses electrical stimulation and pressure to measure pain, initially tested in an animal model. It aims to identify nociceptive pain by observing reactions to pressure and electrical stimuli [41].
6. AlgiScan:for measuring analgesia levels during anesthesia, dolorimeters and similar instruments like algometers and algesiometers apply steady pressure, heat, or electrical stimulation to measure pain threshold and tolerance [42].

Each of these devices offers a unique approach to overcoming the subjective nature of pain assessment, providing valuable tools for both research and clinical practice. They represent significant steps forward in the objective measurement of pain, potentially leading to more personalized and effective pain management strategies.

## Final remarks

The use of local anesthetics/regional techniques in ICUs represents a sophisticated and nuanced area of critical care medicine. With their ability to provide effective pain management with minimal systemic effects, they are invaluable in the ICU setting. However, their use must be carefully balanced against the risks, particularly in the complex and fragile patient population seen in ICUs.

Managing pain effectively is crucial for patients in critical care, especially after surgery or trauma, as it can lessen the stress response and positively impact morbidity and mortality rates. The inadequate treatment of pain in ICU patients often results from limited education, concerns about side effects, and the misuse of medications. Therefore, the involvement of experts in pain management and anesthesiology is frequently essential.

It’s imperative to choose a pain management strategy that aligns with the patient’s specific clinical needs, to provide a personalized pain management plan. The effectiveness of the treatment can be compromised by the selection of inappropriate pain management techniques. A comprehensive approach to pain management, incorporating non-medical, medical, and regional analgesia methods, is often the most effective and necessary. Extending these techniques into the post-surgical period can hasten recovery and facilitate earlier hospital discharge.

Despite their proven safety and efficacy, regional analgesia methods, including neuraxial and peripheral nerve blocks, are not commonly used in critically ill patients. These techniques can minimize the need for opioids and sedatives, reducing the risks of severe side effects associated with these drugs. Proper timing and indication are key to maximizing the benefits of these methods.

The introduction of new technologies, like ultrasonography, has enhanced the safety and efficacy of neuraxial and peripheral nerve blocks, making them viable options even for heavily sedated patients in ICUs. Recent years have seen evolving practices in the use of local anesthetics in ICUs, driven by ongoing research and clinical trials. Areas of particular interest include the use of local anesthetics in managing neuropathic pain in ICU settings, their role in multimodal analgesia strategies, opioid-free analgesia, and their potential systemic effects beyond pain control. Research is also focusing on the development of new local anesthetic agents and formulations that could offer prolonged effects with minimal toxicity.
